# A robotic arm for safe and accurate control of biomedical equipment during COVID-19

**DOI:** 10.1007/s12553-022-00715-1

**Published:** 2023-01-05

**Authors:** Ernesto Iadanza, Giammarco Pasqua, Davide Piaggio, Corrado Caputo, Monica Gherardelli, Leandro Pecchia

**Affiliations:** 1 Department of medical biotechnologies, University of Siena, via Banchi di Sotto 55, Siena, 53100 Tuscany Italy; 2 Department of Information Engineering, University of Florence, Via di Santa Marta 3, Firenze, 50139 Tuscany Italy; 3 School of Engineering, University of Warwick, Library road, Coventry, CV56GB England UK; 4 School of Engineering, Campus Biomedico of Rome, Via Álvaro del Portillo 21, Roma, 00128 Lazio Italy

**Keywords:** Clinical engineering, COVID-19, IPC, Infection Prevention and Control, Robot

## Abstract

**Purpose:**

Hospital facilities and social life, along with the global economy, have been severely challenged by COVID-19 since the World Health Organization (WHO) declared it a pandemic in March 2020. Since then, countless ordinary citizens, as well as healthcare workers, have contracted the virus by just coming into contact with infected surfaces. In order to minimise the risk of getting infected by contact with such surfaces, our study aims to design, prototype, and test a new device able to connect users, such as common citizens, doctors or paramedics, with either common-use interfaces (e.g., lift and snack machine keyboards, traffic light push-buttons) or medical-use interfaces (e.g., any medical equipment keypad)

**Method:**

To this purpose, the device was designed with the help of Unified Modelling Language (UML) schemes, and was informed by a risk analysis, that highlighted some of its essential requirements and specifications. Consequently, the chosen constructive solution of the robotic system, i.e., a robotic-arm structure, was designed and manufactured using computer-aided design and 3D printing.

**Result:**

The final prototype included a properly programmed micro-controller, linked via Bluetooth to a multi-platform mobile phone app, which represents the user interface. The system was then successfully tested on different physical keypads and touch screens. Better performance of the system can be foreseen by introducing improvements in the industrial production phase.

**Conclusion:**

This first prototype paves the way for further research in this area, allowing for better management and preparedness of next pandemic emergencies.

## Introduction

The cause of COVID-19 pandemic, the SARS-CoV-2 virus, is still circulating and differentiating in variants with different levels of aggressiveness and contagiousness [1]. This novel strain of coronavirus has been seriously affecting the daily lives of citizens, worldwide, for approximately the past two years. Back at the onset of the disease, in the midst of the age of globalization, in which people and goods can easily and quickly travel around the world, no one was expecting the advent of a new phase, that of the social distancing and the closing of the borders. Since the start of the pandemic, infection prevention and control (IPC) measures, such as the frequent hand sanitizing with alcohol-based rubs, together with the use of face masks or respirators, have become one of the most effective means to prevent the COVID-19 diffusion. In fact, SARS-CoV-2 can be transmitted via direct (if deposited on people) or indirect (if deposited on objects) contacts and airborne (via droplets and aerosols) routes [2]. Contrasting debates around the actual transmission routes of SARS-CoV-2 are still ongoing in the scientific community [3]. Chin et al. [4] showed that the infectious form of SARS-CoV-2, under laboratory conditions, is traceable on surfaces for several hours depending on the material: less than 3 hours on printing paper or toilet paper, up to 24 hours on wood and textiles, and for up to 3-4 days on smooth surfaces (e.g., steel and plastic). For these reasons, since the onset of COVID-19, more and more individuals have contracted the virus simply by getting in contact with infected surfaces. Certainly, this poses a serious threat for healthcare workers, who come into contact with several potentially infected surfaces during their shifts. Although in late 2020, vaccination programs started, the level of coverage is still far from desired values. Although for high-income and upper-middle income countries, respectively 67.9% and 66.5% of the population has completed the vaccination cycle (with Gibraltar, Portugal, United Arab Emirates, Malta, Singapore, Chile, Cayman Islands, Cuba, Spain, South Korea, and Iceland overshooting 80%) (as of 2 December 2021), for low-income and lower-middle income countries, respectively only 1.4% and 27.9% of the population did. Overall, globally, only 43.8% of the population completed the vaccination cycle (https://ourworldindata.org/grapher/share-people-fully-vaccinated-covid). Moreover, the reduced transmission from breakthrough infections in vaccinated people is still being studied and will require more observational studies following household contacts [3]. Consequently, IPC measures will still be vital to avoid extreme rises in cases and hospitalisations. In light of this, the idea presented in this paper is to design and develop a device able to put in communication the user with a paired electromedical equipment and/or common use interfaces. This novel device is intended to avoid direct contact between users and machine interfaces. Initially, the focus of this study was addressed to three examples of medical devices, i.e., an electrocardiograph, a bedside monitor, and an oxygen concentrator. Afterwards, the focused was shifted to different types of physical keypads and touch screens, because the problem of avoiding contact with contact surfaces is the same and so are the corresponding solutions. In fact, it would be possible to manage electromedical equipment, as well as common-use interfaces, from long distance, through a wireless connection, for instance using Bluetooth or WiFi technologies. Several projects are already available, and many others are still in development, with regard to either tele-operated robots or remote-controlled apparatuses, even in the medical field. For instance, Wang et al. present a review of current researches and future development trends of intelligent robots [5]. In order to provide specificity, the focus must be on a project related to technologies designed to prevent the diffusion of infectious diseases and technologies conceived for remote interaction with buttons, toggles, sliders and so forth. A thorough analysis of the healthcare structures is important to spot possible risks [6]. As reported by “Federazione Nazionale degli Ordini dei Medici e degli Odontoiatri” (FNOMCeO) and “Istituto Superiore di Sanitá” (ISS), in Italy, more than 124,000 healthcare workers have been infected with COVID-19 and, of these, 341 doctors and paramedics died from it (https://portale.fnomceo.it/,https://www.epicentro.iss.it/coronavirus/bollettino/Bollettino-sorveglianza-integrata-COVID-19_10-marzo-2021.pdf.

Hence, a great deal of efforts, by countless research teams, has been focused in developing different devices for the safeguarding of healthcare workers, worldwide. In 2016, a work carried out by Kraft raised the issue of doctors and paramedics’ safety, within an infectious diseases context [7]. In this study, Kraft proposes the use of a tele-operated robot, previously developed by himself and Kraft and Smart [8], and analyses which tasks such a machine should accomplish, for instance, in an Ebola treatment unit. One of the surfacing issues is the problem of visibility, for which the authors performed some trials in both the conditions of visibility and no-visibility between the operator and the patient-simulating person. In the paper by Miseikis et al. [9], a mobile robot platform is designed and described: it is equipped with a multi-function arm, intended for the Human-Robot Interaction (HRI) and for personal care assistant tasks. This robot is able to both assist and interact with people. Lanza et al. propose an improvement of tele-health system features in order to allow patients support, in the same way of human caregivers [10]. Their idea is to provide the patient with requested assistance either in simple clinical assisting scenarios or emergency ones. In the case of a large-scale diffusion, such as the COVID-19 pandemic, their suggestion goes towards the reduction of the contagion risk, by the use of remotely controlled multi-agent architecture for intelligent medical care. The paper by Tavakoli et al. [11] show how robotic and autonomous systems and smart wearables can complement and support healthcare delivery and staff during the COVID-19 pandemic. The main idea is to reduce the infectious disease transmission risks, for frontline healthcare workers, through a safe-distance remote control. Yang et al., within the so-called “Healthcare 4.0” design a novel homecare robotic system (HRS) based on the Cyber-Physical Systems (CPS) [12]. With this new concept of healthcare, all operations are performed remotely. Great technological progresses are required until such a solution could be applicable. Another research carried out by Yang et al. [13] proposes a new tele-robotic system for remote care operation in isolation wards. That system consists of two components, i.e., a teleoperation system and a telepresence system. They hope to reduce infections thanks to the reduction of contact between infected patients and healthcare workers. The review by Zeng et al. summarizes the main applications that robots have as part of different contexts [14]. With regard to researches intended to develop devices for remote interaction with different buttons types, an interesting investigation was carried out by Wang et al. [15]. In this work, authors perform a systematic categorization of buttons and switches, taking over 600 button examples, found at home and workplace environments, as specimens. These researchers designed a subsystem in order to recognise, localise and detect buttons, with high reliability. They realized a tip system able to actuate pull buttons or turn knobs, by remote controlling. In 2010, in Taiwan, Wang and his staff presented a robotic arm prototype, designed on the top of a wheeled robot, able to recognise numbers or signs and to push buttons [16]. In their project, authors involved the use of a micro-camera, placed on the tip of the arm, intended for the image processing and pattern recognition. By means of inverse kinematics, researchers calculated the angle of each robot link in order to successfully press the desired button. Their trials were performed on the elevator panel, the robotic arm was controlled by a computer. We recall that our idea is to project and develop a device able to put in “telecommunication” the user with an electromedical equipment and/or a common use interface. According to European regulations, in order to market a medical device, its design has to be deemed safe by the regulatory bodies of the country under consideration. In this regard, to certify the device as safe, the producer company has to refer to the national versions of international standards, applicable to the concerned device. Compliance with these standards accounts for documentation necessary to demonstrate the actual safety of the designed product. For this reason, it is necessary to apply design principles during the study early stages of the device, paying particular attention to all aspects that guarantee regulatory compliance and certification of the medical device. In order to meet the European requirements it is necessary to adopt a multi-step approach, that is not necessarily a serial process, throughout the cycle, such as [17]: (1) analyze the device to determine which directive/regulation is applicable, (2) identify the applicable essentials requirements list, (3) identify any corresponding harmonized standards, (4) confirm that the device meets the essential requirements/harmonized standards and document the evidence, (5) classify the device, (6) decide on the appropriate conformity assessment procedure, (7) identify and choose a notified body (NB), (8) obtain conformity certifications for the device, (9) establish a declaration of conformity and, lastly, (10) apply for the CE mark.

For medical devices, the International Standards Organization (ISO) established two important standards: ISO 13485 and ISO 14971 [17]. ISO 13485 presents the requirements for a comprehensive medical device designing and manufacturing Quality Management System (QMS). ISO 14971 “Medical devices - Application of risk management to medical devices” was drawn up in 2019 [18]. This standard explains a procedure by which a manufacturer can:

- Identify the hazards and hazard situations associated with medical devices,
- Estimate and evaluate the associated risks,
- Control the risks, and last,
- Monitor the effectiveness of the adopted risk control measures.

As stated in [18], such a process must include: risk analysis, risk evaluation, risk control and production and post-production activities. After those procedures, it is necessary to carry out the risk evaluation (for each identified hazard situations, determining the acceptability or not of those), the risk control option analysis (determining appropriate risk control measures in order to reduce the risks to an acceptable level), the implementation of the risk control measures and the residual risk evaluation (following the risk acceptability defined criteria defined in the risk management plan, drawn up by the manufacturer). Once that is done, the manufacturer must verify the effectiveness of the selected risk control measure, considering also the risk arising from those measures, evaluating then the overall residual risk and reporting everything in the “Risk Management” review.

The International Electrotechnical Commission (IEC), is the leading global organization that prepares and publishes International Standards, Technical Specifications, and so on, with the aim of promoting international cooperation about all the aspects regarding electrical and electronic fields standardization. According to the agreements, the IEC and the ISO collaborate closely in the preparation of standards. Among the documents drawn up by IEC, the 60601-1 is the one concerning medical electrical equipment and medical electrical systems, with particular attention to “general requirements for basic safety and essential performance” [19]. Based on the above standards, we proceeded with the design of the mechanical structure of the aforementioned device. We selected two structural components considered optimal for our purpose: a robotic-arm structure and a x-y-z-axis plotter. 3D models were realised for both the components. Following 3D printing, the robotic-arm was assembled and the actuator motors were programmed via Arduino Mega microcontroller. A multi-platform app, communicating with Arduino, was developed using Ionic 5, Angular and Capacitor frameworks. In accordance to ISO 14971 and IEC 60601-1 regulations, we also carried out a Preliminary Hazard Analysis (PHA) of our device.

## Methods and tools

The aim of our study is to design and develop an automated or robotic system allowing to reduce people’s contact with contaminated surfaces of a selected medical equipment. The automated system has to put in communication the user with the medical equipment and/or common-use surface. Our device is intended to avoid direct contact between users and machine interfaces. Through a wireless connection, for instance using Bluetooth or WiFi technologies, between a remote device, such as a mobile phone app or a programmed joystick, and the designed device, it would be possible to manage a medical equipment, but even common-use interfaces from long distance. Our prototype is composed of two main components:

- An automated robotic system, driven by a micro-controller,
- A mobile phone app, that remotely communicates with the micro-controller.

In light of this, the technologies and tools used for the design and realization of such a prototype are:

- Unified Modeling Language (UML),
- Computer Aided Design (CAD),
- Additive Manufacturing (AM) and Rapid Prototyping (RP),
- Integrated Development Environment (IDE).

### Structural solutions

We selected two structural solutions, considered optimal for our purpose: a 3-axis plotter and a robotic-arm structure.

#### 3-axis plotter

**Fig. 1:**
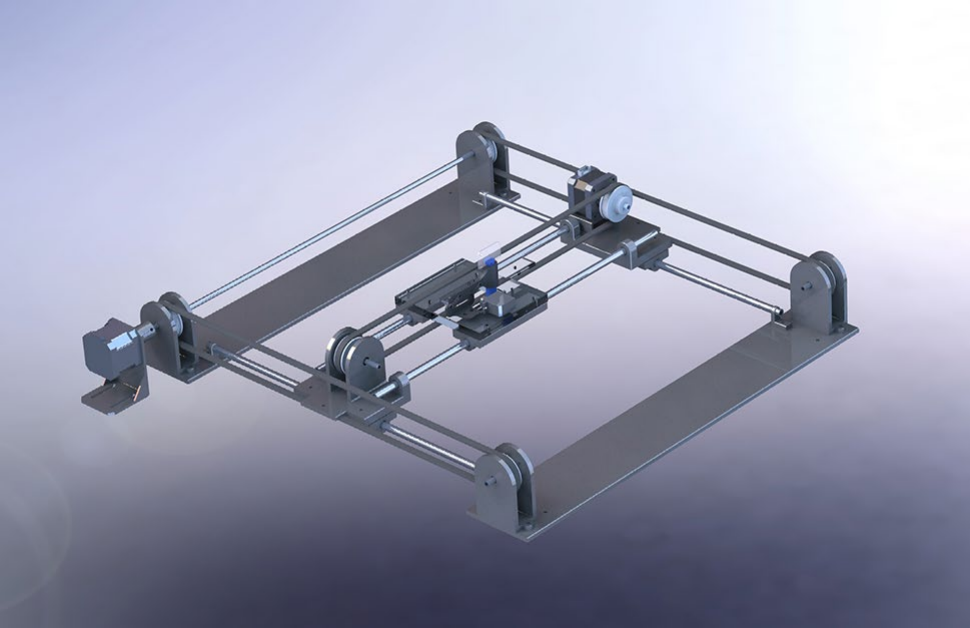
Schematic representation of the 3-axis plotter model

A completely new 3D model was realised for the plotter solution (Fig. 1), using SolidWorks software. Such a model is composed of: a stepper motor and two toothed pulley-belt systems, in order to allow movements on x-axis (horizontal movements) of a linear guide; a stepper motor with a toothed pulley-belt system that moves a linear guide allowing movements on y-axis (horizontal movements); a micro-servo motor allowing movements on z-axis (vertical movements) of an interchangeable stylus, intended for contact with different kind of surfaces (pushbutton, touch screen, soft-touch screen and so on).

#### The robotic-arm model

**Fig. 2:**
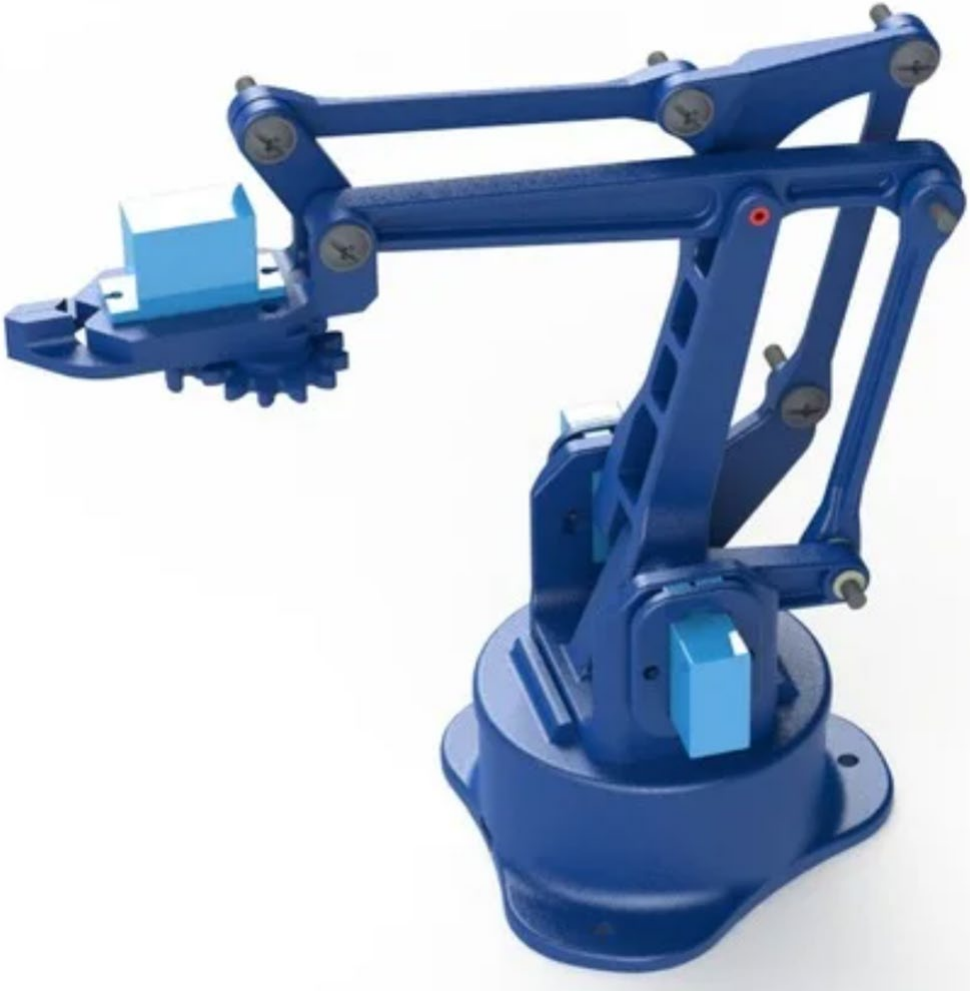
Representation of the 4-DOF 3D printable robotic arm model

The robotic-arm model was chosen as best solution due to its versatility and adaptability to the most diversified situations and environments. To achieve this, at first, we conducted a research of ready-made robotic arms available on the web and we chose a four Degree-of-Freedom (DOF) model, easily purchased in the main online seller and supplier web-pages. Such a model is realised by laser-engraved wood, it is easily assembled and makes use of three micro-servo motors, allowing movements in the space, and an extra micro-servo, allowing the grasping. Then, in order to allow a more reliable and faster testing and to improve the quality of our model, we also carried out a web-search of 3D printable robotic arm models. Among the opensource projects available on the web, we chose the model shown in Fig. 2. Such a model uses four micro-servo motors: three motors for motion through space and one for the grasping function. The robotic-arm was assembled following the 3D printing with Polylactide Acid (PLA) filament. We used PLA, that is a polyester and a thermoplastic polymer, with the characteristic of being biodegradable and derived from renewable sources. PLA is applied to several biomedical fields and is particularly cost-effective, since it results actually easy to produce and dispose, and there are different types in terms of molecular weight, with characteristic modulus of elasticity. The actuator motors were programmed by Arduino Mega microcontroller.

### System design

**Fig. 3:**
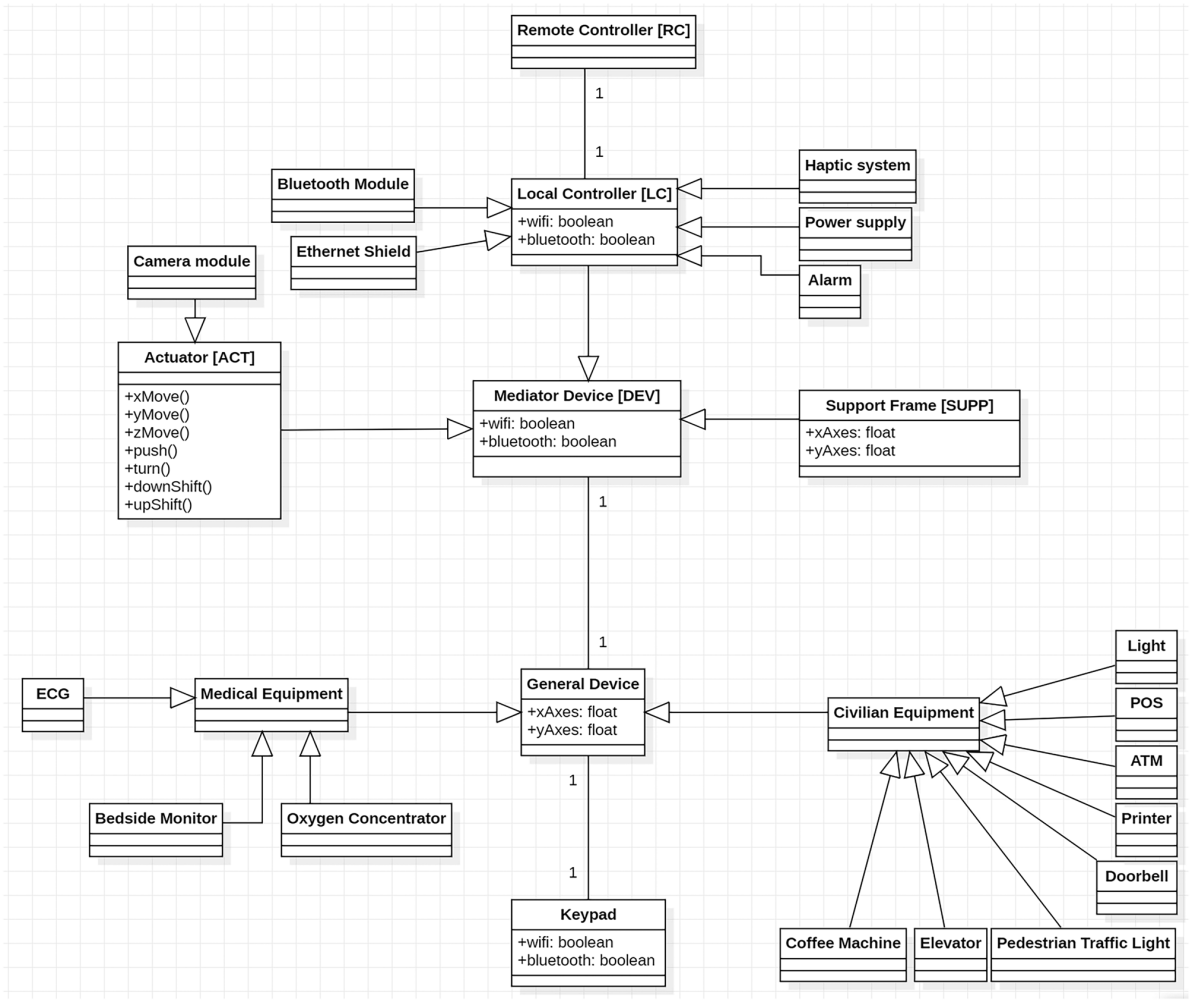
A schematic of the UML class diagram of our device model concept

**Fig. 4:**
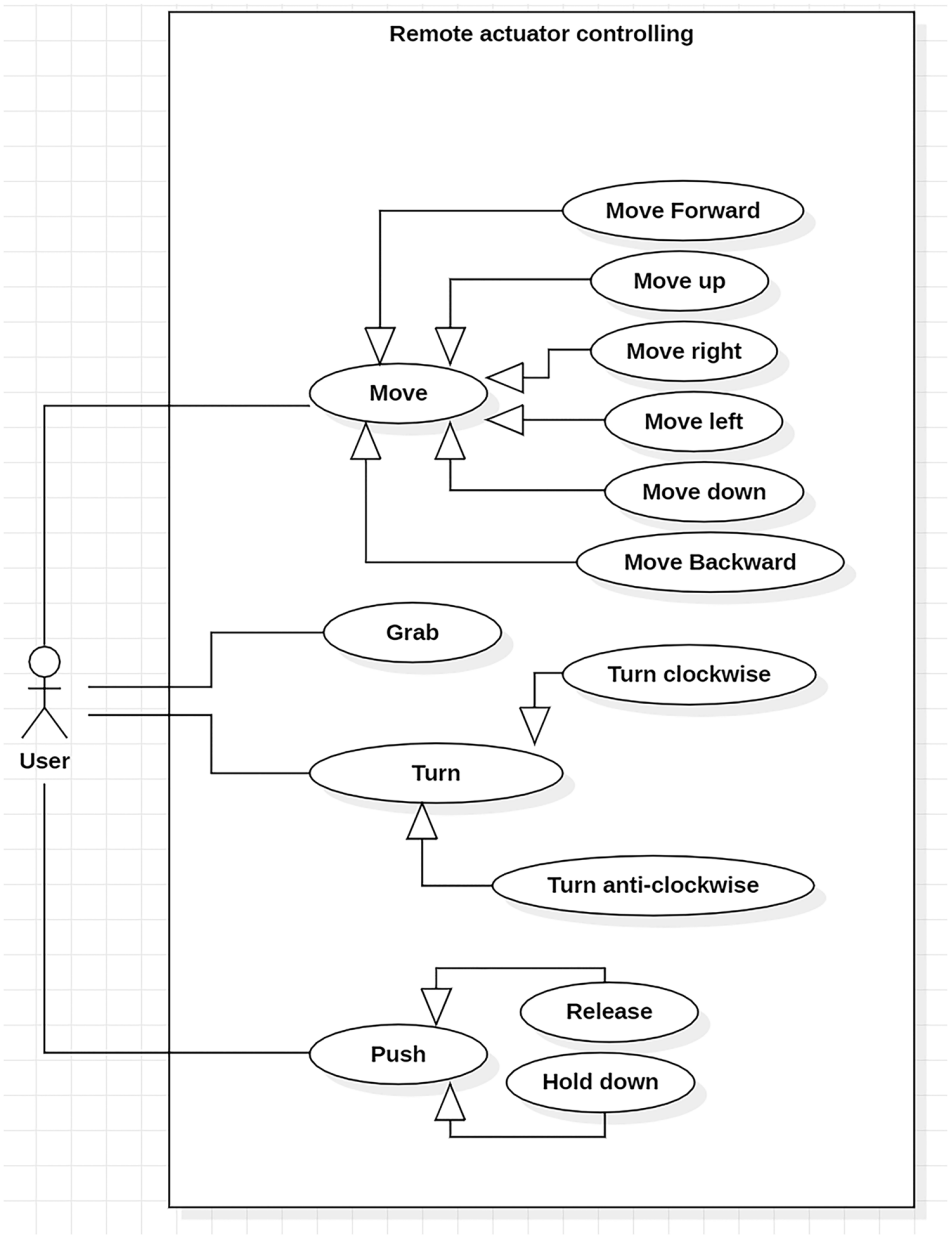
A schematic of the UML use case diagram of our device model concept

**Fig. 5:**
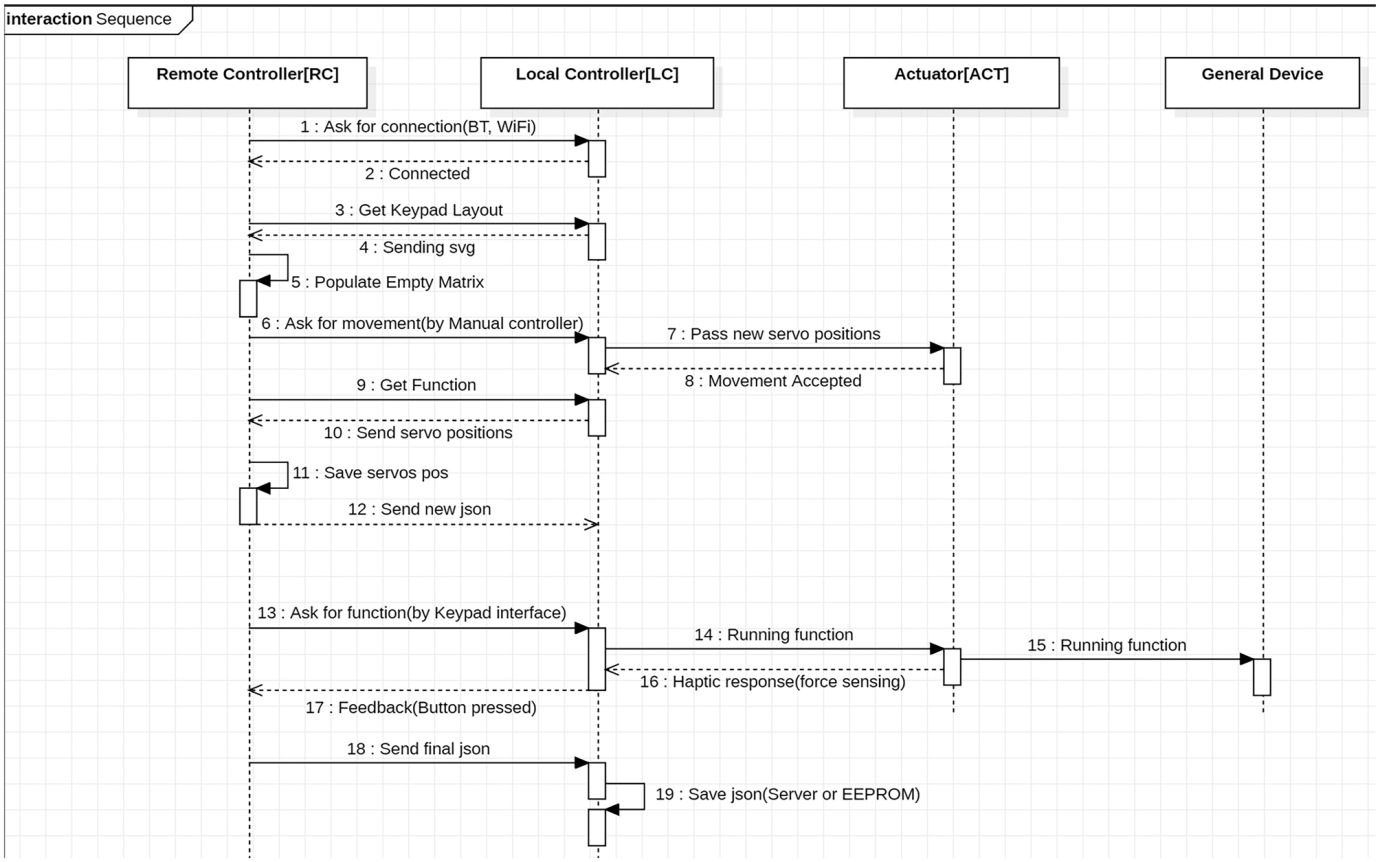
A schematic of the UML interactions sequence diagram of our device model concept

Diagrams created with the UML were created in order to portray the behaviour and structure of our system. UML is a general purpose modelling language with the main aim of defining a standard solution to visualize the way a system has been designed. It is not a programming language, but rather a visual language. As reported by International Business Machines Corporation (IBM) (https://developer.ibm.com/articles/an-introduction-to-uml/), the Object Management Group (OMG) adopted UML as a standard in 1997. It has been managed by OMG ever since. International Organization for Standardization (ISO) published UML as an approved standard in 2005. The language has been revised over the years and is reviewed periodically. UML diagrams are useful, since they help with modelling, design and analysis, and it is possible to categorise them hierarchically. In the following figures we represent three diagrams among the UML diagrams:

- Class diagram (Fig. 3) shows how the different entities (people, things, and data) relate to each other; in other words, it shows the static structures of the system.
- Use Case diagram (Fig. 4) illustrates a unit of functionality provided by the system. Its main purpose is to help development teams visualize the functional requirements of a system, including the relationship of “actors” (human beings who will interact with the system) to essential processes, as well as the relationships among different use cases.
- Interactions Sequence diagram (Fig. 5) shows a detailed flow for a specific use case or even just part of a specific use case. It is almost self-explanatory; it shows the calls between the different objects in their sequence and can show, at a detailed level, different calls to different objects. A sequence diagram has two dimensions: the vertical dimension shows the sequence of messages/calls in the time order that they occur; the horizontal dimension shows the object instances to which the messages are sent (https://developer.ibm.com/articles/an-introduction-to-uml/).

**Fig. 6:**
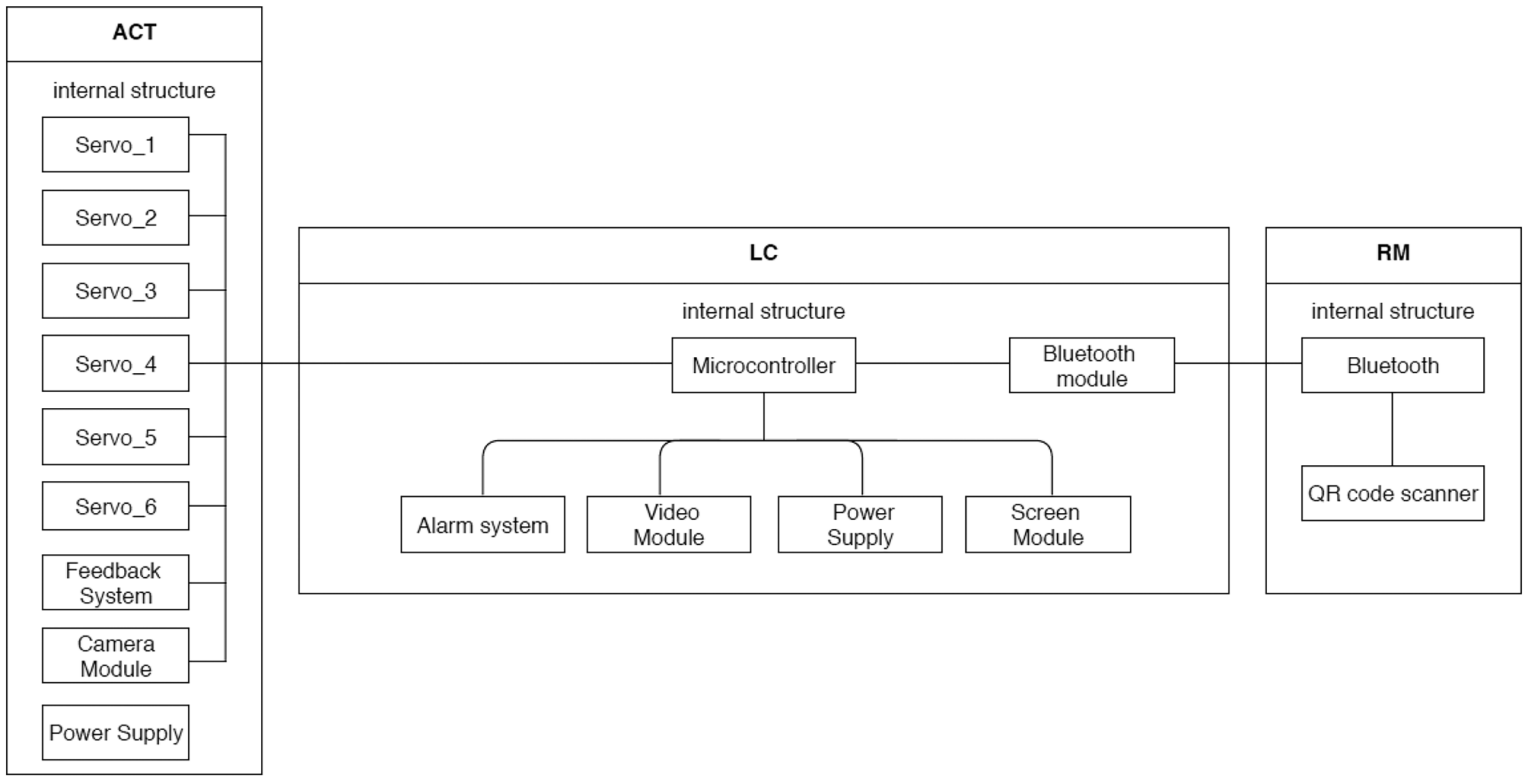
Model-View-Control (MVC) Functional diagram of our model concept

In our class diagram (Fig. 3) we identified four main components: Remote Controller, Local Controller, Mediator Device, General Device. The Remote Controller is intended to be a joystick or a mobile app, easily installed on any mobile phone, to remotely control the Mediator Device, composed of a Support Frame and an Actuator, by means of a Local Controller (the selected micro-controller), which includes few different modules (e.g. Bluetooth module, power supply and so forth). The Mediator Device is the tool directly in contact with the General Device, which might be a medical equipment as well as a civilian equipment, as already stated. In the Use-Case diagram we pinpointed the different tasks that our model concept might accomplish: motion through 3D space for grabbing physical objects (i.e. knobs or sliders), turning objects (such as knobs), and pushing them (physical and non-physical buttons). Finally, in the Interaction Sequence diagram we hypothesized how the functions might be performed and in which logical order. Obviously, all the interactions start from the Remote Controller, come through the Local Controller that deals with the Actuator motion and its interaction with the General Device. In order to make our model concept even clearer, we developed a Model-View-Controller (MVC) functional diagram (Fig. 6). Model-View-Controller is a software design pattern commonly used for developing user interfaces that divides the related program logic into three interconnected elements. This is done to separate internal representations of information from the ways information is presented to and accepted from the user (https://www.artima.com/articles/dci_vision.html). As shown in Fig. 6, we defined all the internal components of each part of our model concept. All the internal components of the actuator, except the power supply, directly communicate with the micro-controller. The Remote Controller communicates with the micro-controller via Bluetooth.

#### The microcontroller

In order to program and control our prototype we decided to use an Arduino Mega 2560 micro-controller (https://www.robotstore.it/rsdocs/documents/Arduino_Mega_overview.pdf). The Arduino Mega is a microcontroller board based on the ATmega1280 microprocessor. It is provided with 54 digital input/output pins: 14 can be used as Pulse Width Modulator (PWM), 16 for analog inputs, and four for Universal Asynchronous Receiver/Transmitter (UART) hardware serial ports. It is equipped with a 16 MHz crystal oscillator, a Universal Serial Bus (USB) connection, a power jack, an “In Circuit Serial Programming” (ICSP) header, together with a reset button. Everything needed to support the microcontroller is onboard. It can boot either connected to a computer, with a USB cable, or powered externally, either with a Alternating-Current(AC)-to-Direct-Current(DC) adapter or a battery (https://www.robotstore.it/rsdocs/documents/Arduino_Mega_overview.pdf).

Such a microcontroller comes with an open-source Integrated Development Environment (IDE), called Arduino Software, or Arduino IDE. This open-source IDE makes it easy to write code and upload it to the board. It runs on Windows, Mac OS X, and Linux. The environment is written in Java and based on Processing and other open-source software (https://www.arduino.cc/en/Main/Software). Arduino company provides everyone with an online version of their open-source IDE (Arduino Web Editor). It allows you to save your sketches in the cloud, having them available from any device and backed up. In such a way, it is possible to always have the most up-to-date version of the IDE without the need to install updates or community generated libraries. In order to work offline, we opted for the offline version of their IDE. Arduino IDE software makes it possible to program Arduino, in order to control four SG90 micro servo motors (https://www.robotstore.it/en/Servo-micro-TowerPro-SG90-9g) by means of a HC-05 Bluetooth module (https://components101.com/sites/default/files/component_datasheet/HC-05%20Datasheet.pdf). Micro servo are tiny and lightweight with high output power. They can provide a rotation of approximately 180 degrees, 90 in each direction. HC-05 Bluetooth module is an easy way to use Bluetooth Serial Port Protocol (SPP) module, designed for transparent wireless serial connection setup. It uses a frequency band centered in 45GHz; the data transfer rate can vary up to 1 Mbps and in range of ten meters. Its communication is via serial communication, which is an easy way to interface with controller or PC.

#### The remote controller

With the aim to remotely control and communicate with the Arduino board, we decided to develop a mobile application (app), that was initially developed using Android Studio, the official Integrated Development Environment (IDE) for Google’s Android operating systems, designed specifically for Android development. Subsequently, for the purpose of making our mobile app available for any mobile devices, we decided to develop a cross-platform app. Cross-platform app development frameworks allow developers to create mobile applications that are compatible with several operating systems. Among the various cross-platform app frameworks, we have analysed some of the most competitive, mature, and top-performing frameworks available today: Xamarin (https://dotnet.microsoft.com/apps/xamarin), React Native (https://reactnative.dev/), Flutter (https://flutter.dev/), Ionic (https://ionicframework.com/docs), Capacitor (https://capacitorjs.com/). Our final decision was Ionic plus Capacitor. The former is an open-source HTML5 SDK that helps developers build native-feeling mobile apps using web technologies like HTML, CSS, and Javascript. The latter is an open source project that uses Progressive Web App (PWA) technology to run modern Web Apps natively on iOS, Android, and the Web, while also providing an interface for accessing Native SDKs and Native APIs on each platform. Thanks to the coupled use of these two frameworks, we were able to develop a cross-platform mobile app, allowing us to remotely control our robotic arm.

#### Remote visualization of the robotic arm work

The actuator is designed to be remotely operated. For instance, with a clear reference to the pandemic situation, it must be possible for the user to control the robotic arm from outside the room where it is placed. This will highly reduce a possible infection risk for the user (e.g. physician or nurse) as well as the need for personal protective equipment. The system has been provided with a camera module to provide the user with a remote visual feedback while operating the robotic arm. For this purpose, we employed a ESP32-CAM module, that is provided with internal Bluetooth and WiFi modules (https://media.digikey.com/pdf/Data%20Sheets/DFRobot%20PDFs/DFR0602_Web.pdf). The app has been implemented with a dedicated code to use the camera module, thus giving the end-user an awareness of which button is really going to be pressed, and reducing the error margin.

### System testing

Once the prototype was finalised, the system was setup and calibrated to perform several ad-hoc tests to evaluate its performance and effectiveness. Such tests were performed on several test panels containing various kinds of buttons in terms of shape and stiffness. In particular, six different interfaces, namely three physical keypads and one touch screen, i.e., Arduino Membrane Switch Module, Casio Calculator Panel, Lexmark Printer Panel, and Lexmark Printer Touchscreen, were selected to this purpose. Tests were performed both with the original robotic tip and an added-on touch pen. Firstly, the overall responsiveness of the different types of buttons was assessed on all the selected interfaces. Secondly, for all the interfaces that were responsive, repeated measures were performed to measure the repeatability and performance of the system. Only the printer touchscreen and the calculator made it to the second part of the tests. Consequently, for testing the robotic arm/printer touchscreen interaction, ten consecutive trials were performed on the same button. As regards the testing of the robotic arm/calculator interaction, ten consecutive trials were performed on eight buttons, i.e., “.”,“,” “2”, “5”, “8”, “=”, “-”, “:”, and “AC”. The number of successful trials was recorded to calculate performance and repeatability.

### Risk management

**Fig. 7:**
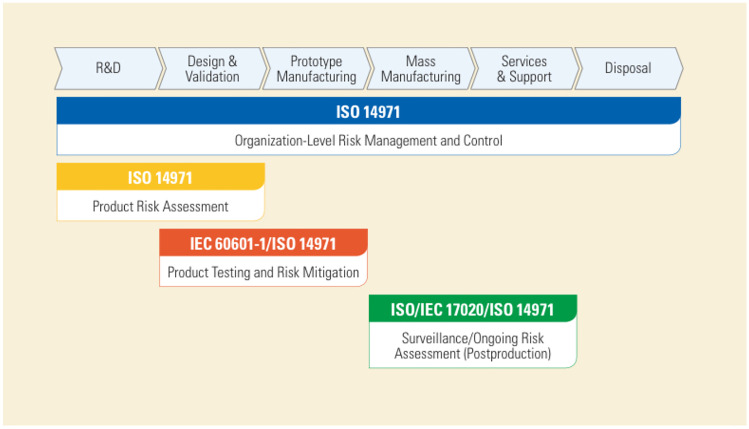
A schematic graph representing regulatory references of each medical device developing steps

Since the proposed device is designed to be also used in medical settings, standards concerning medical devices and electromedical equipment were followed, although a proper risk management according to European regulations was out of the scope of this work. As graphically described in Fig. 7, the ISO 14971 and IEC 60601-1 standards (see Section 1) make up the pillars in designing electromedical equipment.

Indeed these norms provided us with the grounding principles to be followed with reference to “Research and Development” (R &D) and “Design & Validation” (D &V) phases. In accordance with them, we firstly carried out a Preliminary Hazard Analysis (PHA). This analysis is dedicated to analyzing prior experience or knowledge about hazards or failures, in order to identify future and foreseeable hazardous situations, hazards and any kind of events that may result in harm for the end-user (in particular for patient and operator). In order to analyse existing systems or to prioritise hazards, in those cases in which circumstances prevent a more extensive technique from being used, the PHA results to be indeed useful in designing products, processes and facilities. This tool can be used to evaluate the types of hazards for the general product type, then the product class and, finally, the specific product, as well. In early phases of project concept and design development, in which information on design details or operating procedures are still uncertain, PHA is most commonly used. For this reason, it is often put in place as a precursor to further studies. Typically, hazards identified in the PHA are further assessed with other risk management tools (for instance risk ranking and filtering or statistical tools) [20, 21, 22]. For each of the identified hazards, the method includes the following activities:

- Identification of the possibility of the risk event occurring;
- Qualitative evaluation of the extent of possible injury or damage to health that could result;
- Relative ranking of the hazard using a combination of severity and likelihood of occurrence;
- Identification of possible remedial measures.

## Results

**Fig. 8:**
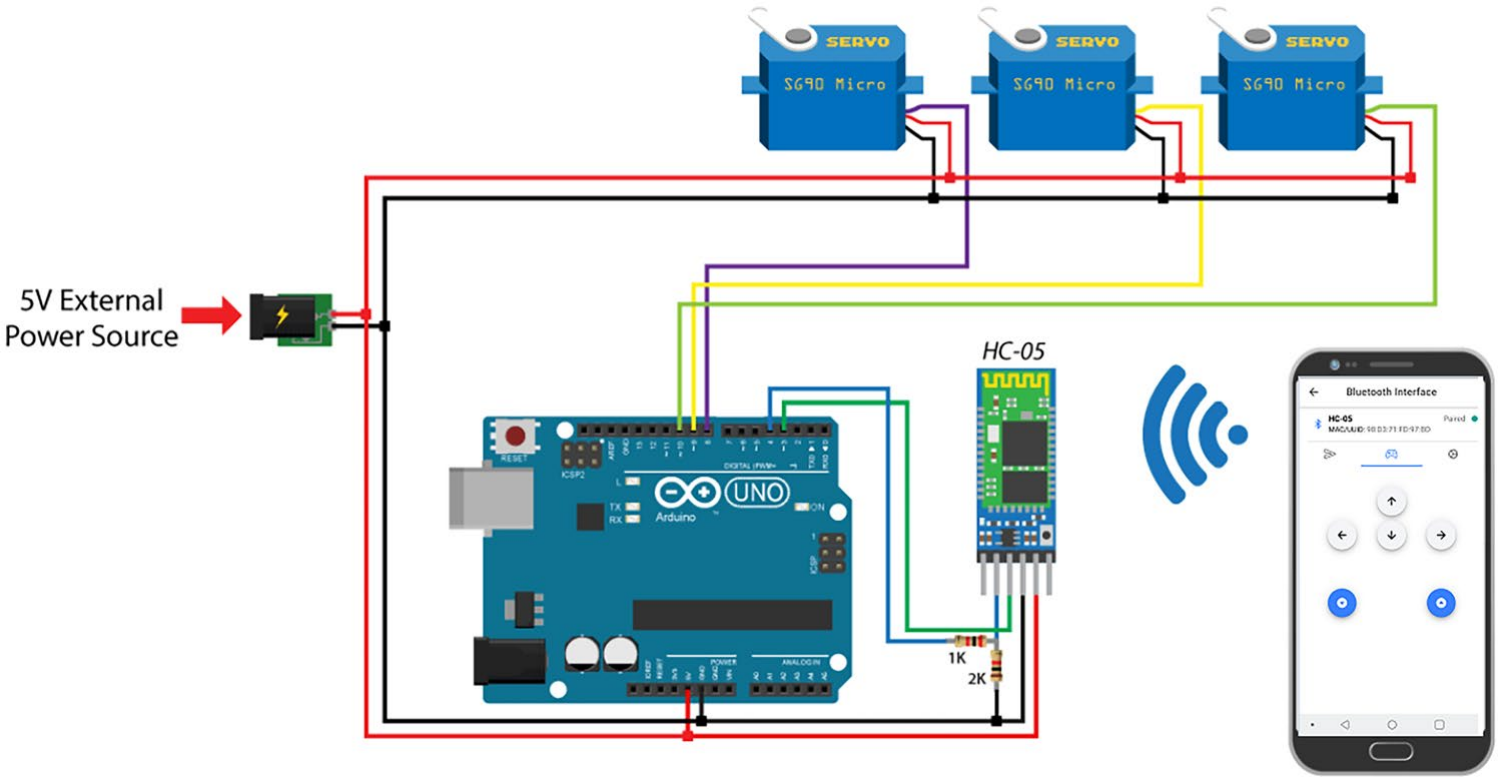
A main hardware configuration sketch of our device (in the sketch is reported Arduino UNO instead of Mega, since it could be used that model as well)

The main configuration of the proposed device is sketched in Fig. 8. With the aim to test the device, other components have been used, such as a Membrane Switch module, a Liquid Crystal Display (LCD) screen module (LCD1602) and a camera module (ESP32-CAM).

### Preliminary hazard analysis

As mentioned above, in order to obtain a product that is safe by design, we carried out a PHA, with reference to the standard for risk management of medical devices (ISO 14971 [18] and to the general norm for electromedical equipment, IEC 60601-1 [19]. An abstract of this PHA is reported in Table 1. As a first step, we analyzed three classes of hazards categories, according to [18]: energy hazards, performance-related hazards and biological and chemical hazards. Then, we focused on the model components: an actuator, a support frame, a local controller and a remote controller. For each of these components, we hypothesized several different hazards for each hazard category. We grouped these hazards by type (e.g. environmental hazards, immunological agents, electromagnetic emission, and more). For each of them we defined five properties: foreseeable sequence of events or hazardous situations, typology of resulting harm (e.g. injury, thermal burns, allergic effect, etc.), severity (by giving a value in a 1-to-5 range), probability of occurrence (by attributing a 1-to-5 value), and possible risk-mitigation interventions (for instance using alarm or choosing suitable materials). The 1-to-5 ranges for both severity and probability of occurrence are reported in Table 2. Following this preliminary analysis, risks can be ranked and filtered. To each risk is associated a value of severity and a probability of occurrence (likelihood). By multiplying the given severity value by the given likelihood value, the risk value is obtained. A risk matrix, like the one in Table 3, can be used to establish a risk acceptability threshold. We chose the threshold value of 9, corresponding to the product of “occasional” (likelihood) and “serious” (severity). Risk values above this value are then classified as not acceptable, and in that case risk reducing measures are required. Risk values between eight and four are considered acceptable, but further investigations are advisable. Risk values below three are accepted. In our PHA are reported some potential risk controls, for each hazard (2. A further risk assessment procedure is necessary to verify if all the risks are acceptable after these control measures, or if new countermeasures should be put in place. A full risk analysis is out of the scope of this article, but will be needed when the proposed prototype will be engineered by a manufacturer, to be CE marked and put on the market [20, 22].

**Table 1:** Abstract of Preliminary Hazard Analysis

**Hazard category**	**PHA Item**	**Hazard Type**	**Foreseeable sequence of events/hazardous situation**	**Harm**	**Severity**	**Probability of harm occurrence**	**Potential risk controls**
Energy Hazards	Actuator	Shape and geometry	Accidental contact with stylus leads to get hurt	Injury	2	3	Stylus design, use instructions/warnings
		Shape and geometry / Materials	The stylus falls down, detaching itself or not from Frame Support	Injury	2	3	Stylus-frame support design avoiding detaching, use instructions/warnings
		High pressure	Contact between Stylus and Machine leads to damage	Damage of stylus/machine	4	3	Stylus material design, haptic system, alarm
		Electrical shock	Stylus transfers electric current to accessible surfaces during operation	Injury	2	2	Stylus design, use instructions/warnings, alarm
		Misuse	User places his own hand between stylus and “machine”	Injury	2	5	Stylus design, use instructions/warnings
		Unsuitable stylus	Unsuitable stylus is used	Stylus and/or ”machine” damaging	3	4	Stylus material design, haptic system, alarm, use instructions/warnings
		Flammability/ Explosiveness	Parts catch on fire/explode	Injury, part damaging	4	2	Stylus design, use instructions/warnings, alarm
	Frame Support	Shape and geometry	Contact with Frame Support leads to get hurt	Injury	2	3	Frame support design, use instructions/warnings
		Shape and geometry / Materials	The Frame support falls down	Injury, sub-assemblies damaging	2	3	Frame support design, use instructions/warnings
		Misuse	Frame support is incorrectly fixed on a “machine”	Injury, part damaging	2	5	Frame support design, use instructions/warnings
	Local Controller	Hot surfaces	Contact with local controller leads to skin burning	Thermal burns	2	4	Alarm, use instructions/warnings
		Shape and geometry	Contact with Local Controller leads to get hurt	Injury	2	3	Local controller design, use instructions/warnings
		Shape and geometry / Materials	The Local Controller falls down	Injury	2	4	Local controller design, use instructions/warnings
		Electrical shock	Local controller transfers electric current to accessible surfaces during operation	Injury	2	3	Local controller design, use instructions/warnings, alarm

**Table 2:** Severity and Probability of Occurrence levels definition

	**Class**	**Definition**
Severity	Negligible (1)	Inconvenience, or temporary discomfort
	Minor (2)	Temporary injury or impairment not requiring professional medical intervention
	Serious (3)	Injury or impairment that requires professional medical intervention
	Critical (4)	Permanent impairment or life-threatening injury
	Catastrophic (5)	Patient or operator death

**Table 3:**
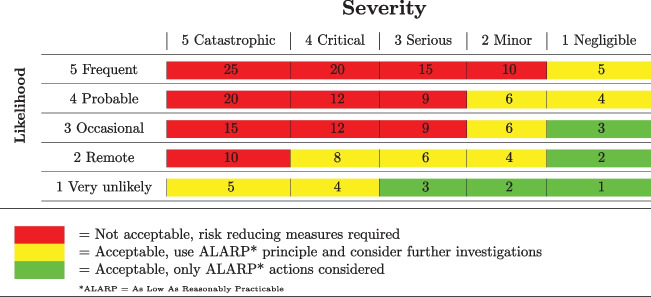
Risk Matrix

### Software

#### The arduino code

The Arduino code is developed in order to program the board and all the modules connected, it is made up of four sections. The first one includes the required libraries and defines variables and constants. The *void setup()* section includes all useful settings required later. The tasks that the Arduino board accomplishes, are programmed in the *void loop()* section. The last part of the code implements the computing functions called by the *void loop()*. The developed code is included in the supplementary materials.

#### The cross-platform mobile app

**Fig. 9:**
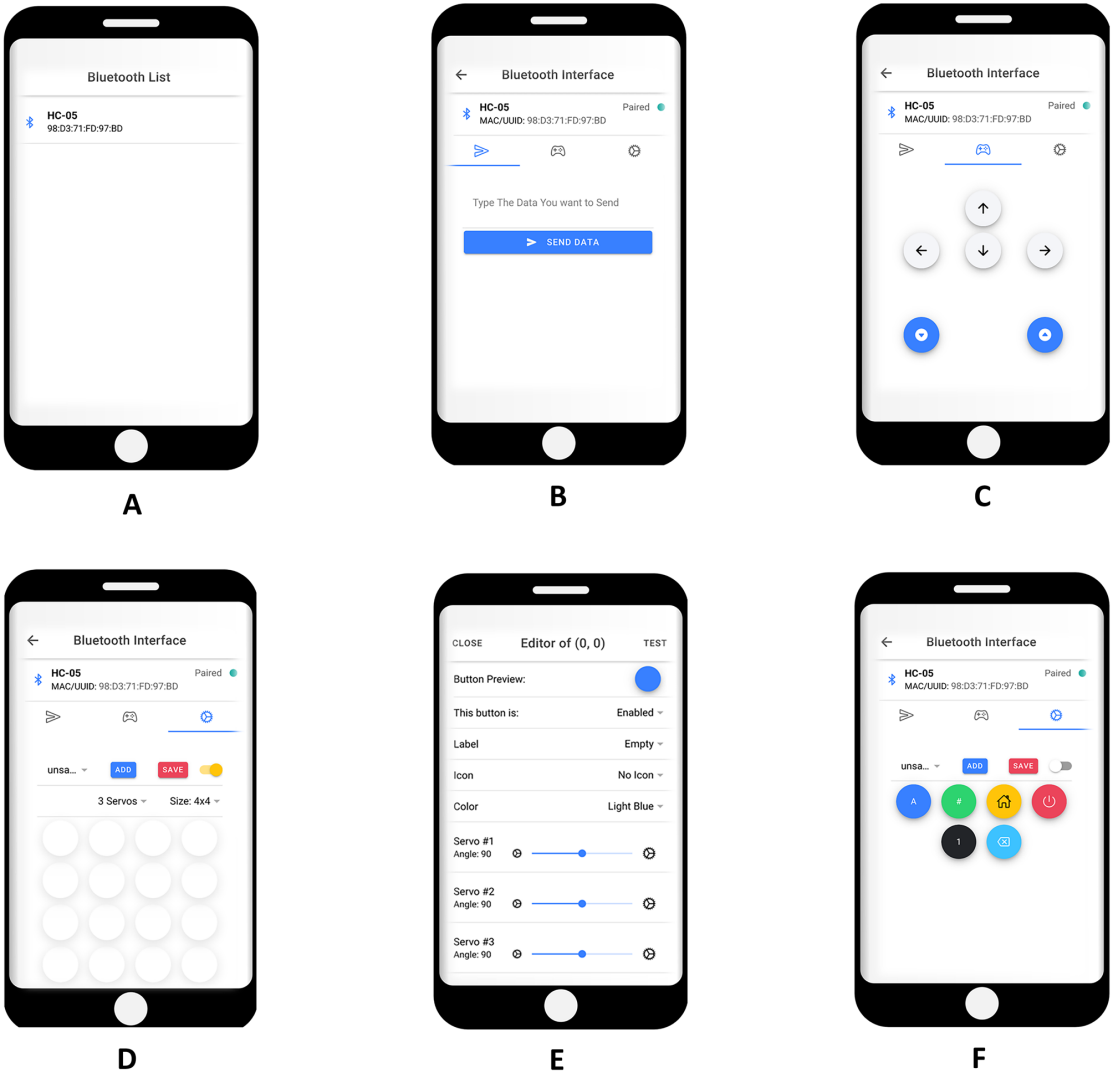
Different tabs of our app: **A** The available Bluetooth modules list page; **B** Typing bar page; **C** Manual Controller tab; **D** Keypad customization interface; **E** Modal page for button features definition; **F** Example of customised keypad

Thanks to the combined use of the above mentioned Ionic and Capacitor frameworks we were able to develop a cross-platform mobile app, that allows to remotely control the robotic arm. The app is multi-tab: after selecting the Bluetooth module to connect to (HC-05 module in our case) the interface shows three tabs. The first one allows to send text by typing on the phone keyboard (meant as a testing tool); the second one is called “Manual Controller” and allows to send commands to Arduino for moving the different servo motors (i.e., up, down, right, left, forward and backward); the last tab graphically reproduces a real keypad, allowing the user to add as many customised keypads as desired, enabling to choose several features such as the dimension of the custom keypad (i.e., 3x3, 4x3, 4x4 etc.) or the number of servo motors to control. It is then possible to activate and customise each button individually. A modal page allows to define a button’s icon (Ionic platform provides several great-looking pre-defined icons), its label (alphanumeric characters or some symbols), and colour, as well as the initial position for each servo motor to control. Once enabled the “customisation enabler” slider, it is possible to “Test” the button, moving servo motors to the defined positions, or to close the page and automatically save the desired button features. Once the keypad setup is finished, it is possible to “Save” the custom keypad and then to “Add” a new one. Once finished, the “customisation enabler” slider can be switched off and the selected keypad is ready to be selected from a drop-down menu and used. The following Fig. 9 shows all the different tabs of the developed cross-platform app.

### Tests

In order to test and evaluate performances and effectiveness of our prototype, we carried out the system setup and calibration and performed some trials using test panels containing buttons of various types, shapes, and stiffness. We employed six different interfaces which our arm had to interact with: three physical keypads and three touch screens.

#### Robotic arm and app setup

To apply the method to a new environment, a manual setup procedure must be performed to populate a database of button panels, using the above described procedure. The process is relatively fast and the environment is minimally altered. For example, completing annotation, calibration, and information entry for a general device panel with 16 to 20 buttons takes about 20 minutes. Servo motors position setting requires more time, but the whole setup of a new keypad takes less than one hour. The procedure consists of the following steps:

- Definition and setting up of robotic arm position relative to the keypad being tested;
- Setting up of an interface resembling the real keypad, on the mobile app;
- Definition of servo motors positions for each button.

**Environmental Annotation and Calibration** - At first, we fixed the robot arm base on a platform: its layout allows to easily fix the support frame using some screws. The center of the base has been fixed at a distance of 20cm from the wall containing the keypads to be operated. The base was positioned at 47cm above ground, keypads at 64cm. The robotic arm maximum extension is 23cm outward, allowing the robot to press buttons placed on a 20cm-radius semi-sphere, being it moved by micro-servo motors with 180-degrees motion ability (90 degree in both directions). By testing with these settings, it resulted that this robot is able to frontally push buttons placed in a range between 46cm to 62cm above the ground (approximately equal horizontal range). 

**Keypad customization on the mobile app** - Once all the spatial settings have been configured, we proceeded to replicate the test keypads on the dedicated mobile app tab. As already stated, this step is fairly quick, taking roughly one hour to replicate the calculator panel, composed of 20 buttons (smallest buttons were excluded), and a little less for the other simpler panels. To set the positions of each micro-servo motors for each button, we made use of the manual controller app tab that aided to find them easily. 

**Fig. 10:**
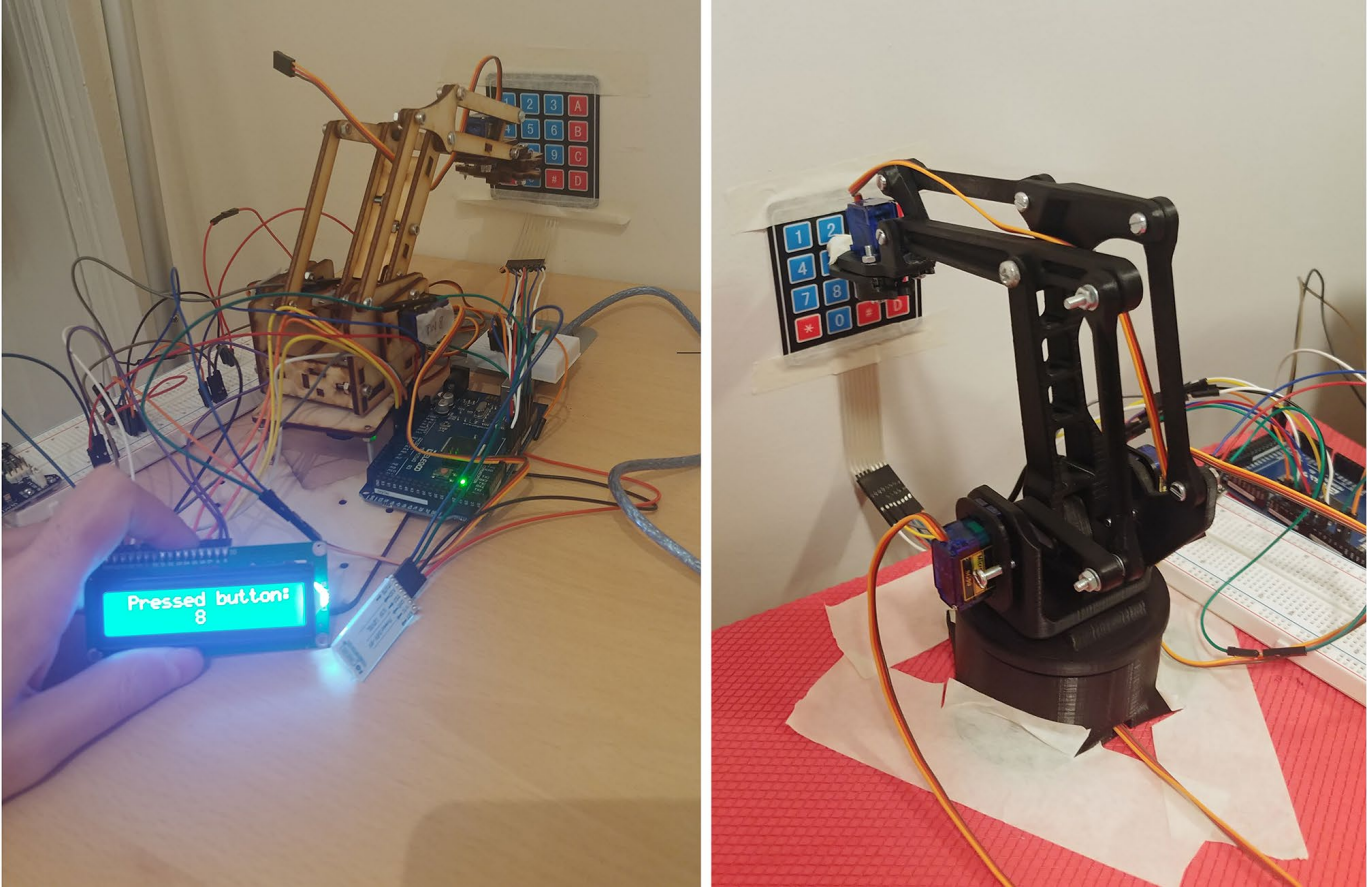
Photos of tests with the switch membrane module of Arduino and both the wood and the PLA models

**Tests of pushing buttons and related results** - Upon completing the setup phase, we proceeded testing the five different keypads as anticipated in the methods. First of all, we tested the switch membrane module of Arduino. With the exception of few sporadic events, our robot was not able to press any buttons and the repeatability of the task was inconsistent. The keypad button stiffness is too high to allow the robot to complete the required function. Moreover, our model is not rigid enough in comparison to such a keypad and it tends to slip on it. Those tests have been performed using both the wooden 3D robotic arm, and the PLA one, as it is possible to see in Fig. 10.

After that, we tested a printer panel (printer model Lexmark CS727). Similarly to the previous keypad, buttons of that panel are too stiff for our robotic arm that, once again, tends to slip on it. No buttons have been pushed by the robotic arm during tests. Differently, the robot was well capable to press the touch screen of the printer without any issues, showing a sensitive and responsive behaviour. We queried the robot to press the same button ten times and it worked correctly every time (success rate of 100%). Considering that the actuator probe is suited for grabbing and holding small objects but it is not appropriate as pointer, meant to press any kind of buttons, we mounted a rubber “touch pen” on the robotic arm clamp. The interaction between the tip and the interfaces (calculator keypad and printer touch screen) seemed to qualitatively improve. Finally, we tested the ability to operate a calculator panel. The button stiffness is such that the robotic arm could readily and effectively push those buttons. We tested the following buttons: “.”, “2”, “5”, “8”, “=”, “-”, “:” “AC”. Each button was pressed ten times in a row, for a total of 80 trials. These trials have been performed successfully 76 times over 80, with a repeatability/success rate equivalent to 95%. The robotic arm was able to press buttons with and without the pen mounted on.

## Discussions

The system prototype (app and robotic arm) presented in this manuscript was developed by integrating different engineering methods. This system prototype is conceived for decreasing the contact events with common surfaces such as the interfaces of medical devices and civilian ones (e.g., elevator panels). In fact, it is designed in a way so that the users can interface themselves with such devices, via our mobile app, installed on their smartphone, which communicates with our robotic arm that is installed on the device/surface of interest. This communication can rely either on Bluetooth or WiFi. This long-distance interaction is enhanced by the camera module, mounted on the robotic arm, which allows for visual feedback of the button pressed on the interface by the tip. This aims at improving the capability of pressing a button and the robot intrinsic safety (to ensure the kinematic movement was achieved or that the arm would not move in a self destructive manner). The performance of the system was also enhanced by adding a “touch-pen stylus” as a tip of the robotic arm. Using such a system, the likelihood of getting indirectly (i.e., because deposited on surfaces) infected with SARS-CoV-2 or other viruses and/or bacteria is extremely reduced. To the authors’ best knowledge, this is the first attempt of its kind. In literature, in fact, there are several descriptions of robots for different healthcare and infection, prevention, and control purposes, spanning from their use for automatised disinfection, to the control of hand sanitising practices, and to the management of patients. In particular, the robots described by Kraft [7], Miseikis et al. [9], Lanza et al. [10] focus on the assistance of people and patients, and on human interaction. The robot introduced by Wang et al. [16] is only aimed at working on elevators and not at infection, prevention, and control purposes, and uses different principles from ours. The robot introduced by Yang et al. [13] brings together robotics and telemedicine and is designed to be used in isolation wards for the visit of patients. Our robot was designed bearing in mind the medical device regulations and their strict requirements about risk analysis, evaluation and management, as duly explained in ISO 13485 (Quality Management Systems) and ISO 14971 (Medical devices - Application of risk management to medical devices). For the low-level and low-cost technologies that we have employed, our results were satisfactory. Better results could be gained just improving the underlying technologies. The cross-platform mobile app results to work properly and to communicate without any issues with Arduino, via Bluetooth, but it could be easily improved with further features. Optimization measures have been carried out in order to improve the prototype effectiveness. A “touch-pen stylus” have been mounted on the robotic arm with the aim of improving the capability of pressing a button. With reference to future improvements and development, we can summarize some possibilities. Regarding the structure of the system, it could be appropriate to design and develop a linear guide on which to mount the robot base, in order to allow interaction with larger interfaces or horizontal-emphasis interface. Moreover, a bigger robotic arm model, requiring proper servo and stepper motors (with higher torque powers) could improve the interaction range. Higher DOF would be advisable with the aim of developing further functions, such as that of turning a knob, moving a slider, switching a toggle or pulling a lever. For this purpose it could be useful to design and develop a custom robotic tip capable of accomplishing those mentioned multiple functions, with the same efficacy. With reference to the remote control of the system, it could be appropriate to program a more performing micro-controller, for instance STM32 Nucleo boards, in order to provide improved UI experience. Even the development of a webserver, in which to save different keypad replicates and from which to retrieve them via the mobile app, would add value to the whole system. In this case, the development of a specific subsystem, in the mobile app, would be necessary to communicate with the webserver.

## Conclusions

The current COVID-19 pandemic, characterized by remarkable severity and diffusion, has been without equal in modern history. The tragic times caused by COVID-19, showed how crucial, ingenious, and resilient are healthcare and essential worker, worldwide. In light of this, with this work, we brought forward a project dealing with a robotic system that could immediately be useful to the healthcare system, healthcare workers, and society, during the current and likely future pandemics/epidemics. Indeed, the safe and efficient progress of healthcare does not only rely on medicine, but also on engineering, science and technology. This paper illustrates the design of a device conceived for avoiding direct contact between users and interfaces of selected medical equipment and/or of interfaces of civilian use. Our prototype is composed of two main components: an automated/robotic system, driven by a micro-controller, and a mobile phone app that remotely communicates with the micro-controller. Therefore, it is possible to manage the medical equipment or interface for civilian use from long distance through a wireless connection, using Bluetooth or WiFi technologies. The point of strength of our approach, also presented in this paper, is the PHA that we carried out on the conceived device and that guided our design phase. This step was essential to be compliant with the stringent requirements for risk analysis, assessment, and management of ISO 13485, ISO 14971, and of the European Medical Device Regulations (2017/745) (https://eur-lex.europa.eu/legal-content/EN/TXT/?uri=CELEX%3A32017R0745). Such regulations and standards, specifically the latter, would have to be applied in case our system were to be used together with a medical device to pursue a medical intended purpose.
